# Mapping completeness

**DOI:** 10.1107/S2052252521011106

**Published:** 2021-11-01

**Authors:** Martin Kunz

**Affiliations:** a LBNL, 1 Cyclotron Rd, Berkeley, CA 94720, USA

**Keywords:** diamond anvil cell, high-pressure XRD, X-ray data completeness

## Abstract

Tchoń & Makal [ *IUCrJ* (2021), **8**, 1006–1017] use numerical simulations to explore the dependence of data completeness on crystal orientation, X-ray energy and diamond anvil cell geometry for high-pressure diffraction experiments. Their completeness heat maps for different Laue classes can be used to guide optimization of high-pressure single-crystal diffraction experiments.

Diamond anvil cells (DACs) are simple devices that allow experiments involving electromagnetic radiation as a probe to be performed in the visible, IR and hard X-ray range at pressures between ~10^8^ and 10^11^ Pa. Like almost anything of significance in the realm of DACs, their use for single-crystal X-ray diffraction was pioneered by Bill Bassett in Cornell in the 1970s (Merrill & Bassett, 1974 ▸). These simple devices allowed a pressure dimension to be added to crystallography for the first time, thus providing an invaluable tool in the quest to experimentally explore the nature of the chemical bond in chemistry, physics and mineralogy. Not surprisingly, DACs found immediate acceptance and countless groups all over the world adopted this simple but powerful concept for high-quality structural studies at high pressures, which led to further refinements of DAC design and use (Allan *et al.*, 1996 ▸; Finger & King, 1978 ▸; Kudoh *et al.*, 1986 ▸; Miletich *et al.*, 2000 ▸). For high-pressure single-crystal diffraction data to be of sufficient quality to enable accurate bond lengths, bond angles and displacement parameters to be extracted, a series of experimental artifacts and difficulties specific to DACs have to be overcome. These include precise crystal centering with reduced optical access (King & Finger, 1979 ▸), diamond absorption (Angel *et al.*, 2000 ▸), gasket shadowing (Katrusiak, 2004 ▸) and intensity modulation through diamond anvil diffractions (Loveday *et al.*, 1990 ▸).

An intrinsic limitation on data that comes with single-crystal diffraction in ancillary equipment is the restriction of accessible reciprocal space, thus making the collection of a complete set of unique diffraction intensities either difficult or impossible. In the case of DACs, this restriction was quantified for the first time, true to my comment above, by Bill Bassett (Merrill & Bassett, 1974 ▸) who found that the accessible reciprocal space forms a toroidal annulus (Fig. 1 ▸) whose volume is determined by the accessible *q*-range (*i.e.* wavelength and maximum diffraction angle) as well as the conical opening angle α of the DAC. Consequently, attempts to improve the completeness of high-pressure diffraction data were primarily directed towards improved DAC design (Allan *et al.*, 1996 ▸; Kantor *et al.*, 2012 ▸; Miletich *et al.*, 2000 ▸) and improved diamond design (Boehler & De Hantsetters, 2004 ▸) to maximize the effective opening angle α and thus the toroidal subspace of the reciprocal lattice (Fig. 1 ▸).

One important aspect that crucially affects data completeness and is complementary to hardware development, and which was also already mentioned in Merrill & Bassett (1974 ▸), is the orientation of the sample crystal relative to the DAC geometry. This has never been fully quantified in a systematic way. Tchoń & Makal (2021 ▸) in this issue of **IUCrJ** , close this gap with a comprehensive and thorough study that systematically assesses the relative contributions of sample orientation, X-ray energy and DAC opening angle to the completeness of the diffraction data as a function of Laue class (symmetry). The level of completeness for a given experimental arrangement (*i.e.* combination of 2θ_max_, X-ray energy/wavelength, sample Laue class, DAC opening angle α and crystal orientation relative to DAC geometry) is quantified by a ‘Potency’ *P* that is the ratio between the ‘classical’ completeness as defined in *International Tables Volume G* and an ‘applicable’ completeness which depends on the experimental set-up (*i.e.* accessible reciprocal space and sample orientation). The results are visualized for a series of important Laue classes and in-house experimental conditions in the form of heat maps projected on a unit sphere. These heat maps are of real immediate practical value for any high-pressure crystallographer in that the maps allow them to get a quick sense of the sensitivity of their experiment to crystal orientation. They furthermore allow for a rough quantitative estimate of the best crystal orientation. An obvious and important relatively low hanging next step based on this work is to create an app to assess the Potency (*i.e.* achievable completeness) of a given experimental set-up and make the app available to the crystallography community as an on-line tool. And the equally logical but harder to achieve step after that is to develop a set of micromanipulation tools in conjunction with orientation photographs of the sample to achieve the perfect sample orientation of a ~100-µm crystal on a diamond culet of slightly larger dimensions. This for the time being is still reliant on the skills and calm hands of invaluable graduate students and postdocs.

Besides these practical aspects, the results of the numerical simulations also demonstrate – somewhat surprisingly – that the energy range chosen is only of secondary importance for the completeness of the dataset. This conclusion may well be biased by the limited energy range considered (20 keV versus 22 keV), as those two energies are relevant for in-house laboratory X-ray sources, and will probably have to be revisited once synchrotron-accessible energy ranges (up to 40 keV, ~0.3 Å) are taken into consideration.

## Figures and Tables

**Figure 1:**
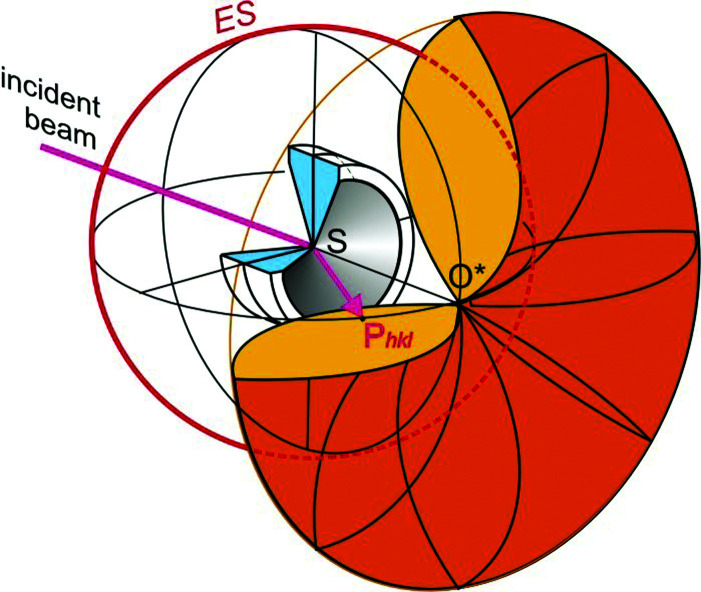
Illustration of accessible reciprocal space through a diamond anvil cell with a conical opening angle α. The colored toroidal volume *V*_DAC_ is given by *V*_DAC_ = 4π/λ^3^[sin α(α — sin α cos α)] (Merrill & Bassett, 1974 ▸; Miletich *et al.*, 2000 ▸). Tchoń & Makal (2021 ▸) quantify systematically how a sample crystal of given symmetry needs to be oriented relative to the DAC in order to maximize the number of independent reflections in the toroidal volume. Figure modified after Miletich *et al.* (2000 ▸).
